# Characterizing neutral genomic diversity and selection signatures in indigenous populations of Moroccan goats (*Capra hircus*) using WGS data

**DOI:** 10.3389/fgene.2015.00107

**Published:** 2015-04-07

**Authors:** Badr Benjelloun, Florian J. Alberto, Ian Streeter, Frédéric Boyer, Eric Coissac, Sylvie Stucki, Mohammed BenBati, Mustapha Ibnelbachyr, Mouad Chentouf, Abdelmajid Bechchari, Kevin Leempoel, Adriana Alberti, Stefan Engelen, Abdelkader Chikhi, Laura Clarke, Paul Flicek, Stéphane Joost, Pierre Taberlet, François Pompanon, NextGen Consortium

**Affiliations:** ^1^Laboratoire d'Ecologie Alpine, Université Grenoble-AlpesGrenoble, France; ^2^Laboratoire d'Ecologie Alpine, Centre National de la Recherche ScientifiqueGrenoble, France; ^3^National Institute of Agronomic Research (INRA Maroc), Regional Centre of Agronomic ResearchBeni-Mellal, Morocco; ^4^European Molecular Biology Laboratory, European Bioinformatics InstituteHinxton, UK; ^5^Laboratory of Geographic Information Systems (LASIG), School of Civil and Environmental Engineering (ENAC), École Polytechnique Fédérale de LausanneLausanne, Switzerland; ^6^Regional Centre of Agronomic Research Errachidia, National Institute of Agronomic Research (INRA Maroc)Errachidia, Morocco; ^7^Regional Centre of Agronomic Research Tangier, National Institute of Agronomic Research (INRA Maroc)Tangier, Morocco; ^8^Regional Centre of Agronomic Research Oujda, National Institute of Agronomic Research (INRA Maroc)Oujda, Morocco; ^9^Centre National de Séquençage, CEA-Institut de GénomiqueGenoscope, Évry, France; ^10^NextGen Consortiumhttp://nextgen.epfl.ch/

**Keywords:** *Capra hircus*, WGS, genomic diversity, population genomics, selection signatures, indigenous populations, Morocco

## Abstract

Since the time of their domestication, goats (*Capra hircus*) have evolved in a large variety of locally adapted populations in response to different human and environmental pressures. In the present era, many indigenous populations are threatened with extinction due to their substitution by cosmopolitan breeds, while they might represent highly valuable genomic resources. It is thus crucial to characterize the neutral and adaptive genetic diversity of indigenous populations. A fine characterization of whole genome variation in farm animals is now possible by using new sequencing technologies. We sequenced the complete genome at 12× coverage of 44 goats geographically representative of the three phenotypically distinct indigenous populations in Morocco. The study of mitochondrial genomes showed a high diversity exclusively restricted to the haplogroup A. The 44 nuclear genomes showed a very high diversity (24 million variants) associated with low linkage disequilibrium. The overall genetic diversity was weakly structured according to geography and phenotypes. When looking for signals of positive selection in each population we identified many candidate genes, several of which gave insights into the metabolic pathways or biological processes involved in the adaptation to local conditions (e.g., panting in warm/desert conditions). This study highlights the interest of WGS data to characterize livestock genomic diversity. It illustrates the valuable genetic richness present in indigenous populations that have to be sustainably managed and may represent valuable genetic resources for the long-term preservation of the species.

## Introduction

Livestock species play a major socio-economic role in the world since they provide many goods and services to human populations. Goats (*Capra hircus*) in particular are one of the more important livestock species, because of their high potential of adaptation to harsh environments. They had a worldwide population of about 1006 million in 2013 (http://faostat3.fao.org/browse/Q/QA/E) and, together with cattle and sheep, they represent the most important source of meat, milk, and skin.

Goats are considered to be the first ungulate to be domesticated, about 10,500 to 9900 years ago near the Fertile Crescent (Zeder, 2005; Naderi et al., 2008). Following human migrations and trade routes, goats rapidly spread over the rest of the world, mainly in Eurasia and Africa (Taberlet et al., 2008; Tresset and Vigne, 2011). During this expansion, they became adapted to different climatic conditions and husbandry practices. In response to these environmental and anthropic selection pressures, a large variety of locally-adapted populations emerged. These populations were managed in a traditional way, *i.e*., with moderate selection for traits of interest and reproduction allowing important gene flows among them, thus maintaining high levels of phenotypic diversity (Taberlet et al., 2008). However, the rise of the breed concept during mid-1800s (Porter, 2002), and its application to husbandry practices, led to the creation of well-defined breeds. This process aimed at standardizing phenotypic traits mainly associated with morphological aspects (e.g., coat color). Selection of animals for these traits was generally moderated, while crossing among different phenotypes was reduced (Taberlet et al., 2008). More recently, since mid-1900s, industrial breeding has become more widespread, backed by the progress of husbandry practices including the introduction of artificial insemination, embryo transfer, the improvements in feed technology and the use of vaccines and therapeutics against endemic diseases. This has led breeders to progressively substitute the many locally-adapted indigenous breeds for very few highly productive cosmopolitan ones for short-term economic reasons (Taberlet et al., 2008). Thus, FAO in 2013 estimated that 18% of local goat breeds over the world were threatened or already extinct (http://faostat.fao.org/). Consequently, a part of the highly valuable genetic resources captured from the wilds and gradually accumulated over 98% of their common history with humans is now threatened (Taberlet et al., 2008).

Thus, it appears crucial to assess the genetic resources of indigenous populations in order to manage them sustainably and to propose zootechnical approaches that take into account the preservation of genetic resources. This might be critical in the current context of global environmental changes. To accurately characterize genetic resources, it is necessary to access variation data across the whole genome. This would allow the identification of alleles related to contrasted environmental conditions and those potentially playing an adaptive role. Recent progress in sequencing technologies has opened new perspectives toward the magnitude of genetic analysis that is possible. Sequencing cost and time have dramatically decreased (Snyder et al., 2010) and it is now possible to obtain Whole Genome Sequencing (WGS) data for several dozen individuals, which allows access to variation data sets of the whole genome (Altshuler et al., 2012; Kidd et al., 2012). It is thus possible to combine WGS data and population genomic approaches to characterize neutral and adaptive variation in an unprecedented way. This allows an accurate characterisation of genetic resources and their geographic distribution. The Moroccan territory represents an ideal case-study for evaluating the potential of indigenous breeds for constituting neutral and adaptive genomic resources. Despite the massive introduction of “cosmopolitan” breeds to improve goat milk production in some areas, indigenous populations still represent about 95% of Moroccan goats. This proportion has been continually decreasing and this could lead in a mid-long term to the complete absorption of some indigenous populations by cosmopolitan breeds. In Morocco there are more than 6.2 Million goats (http://faostat3.fao.org/browse/Q/QA/E). Direct anthropic selection was relatively modest and until recently it was difficult to distinguish well-defined breeds. However, several phenotypic groups displaying specific morphological and adaptive characteristics have been identified. They will be referred hereafter here as populations. The three major groups are: (i) the Black goats with three sub-populations that have been recently officially recognized (Atlas, Barcha and Ghazalia), (ii) the Draa population, (iii) and the Northern population. Besides these three main populations/breeds, the major proportion of Moroccan goats presents intermediate phenotypes and non-recognized local populations. The Black population is characterized by its dark color, long hair, a low water turnover and thus good resistance to water stress (Hossainihilali et al., 1993). It presents a good acclimation to various environmental conditions in Morocco (from the Eastern plateaus to Atlas Mountains and the Souss valley more in the South). The Northern population displays some phenotypic similarities with Spanish breeds such as the Murciana-Granadina, Malaguena or Andalusia breeds (Benlekhal and Tazi, 1996). It is bred for milk and meat production although it presents a lower level of production than cosmopolitan industrial dairy breeds (Analla and Serradilla, 1997). It shows a substantial reproductive seasonality related to photoperiod variation (Chentouf et al., 2011). Following an extensive breeding system, it is the preferred breed to be raised in the harsh mountains of the extreme North of Morocco with oceanic influence and a milder climate. The Draa population is bred in the oasis in Southern Morocco, which is characterized by arid/desert climate conditions. Its water turnover is low compared to European goat breeds studied in similar environments. The Draa goat also has the ability to maintain an unchanged food intake during periods of water deprivation (Hossaini-Hilali and Mouslih, 2002). It displays relatively higher performances of reproduction (i.e., prolificacy, earliness; Ibnelbachyr et al., 2014) and hornless individuals represent about 54.1% of the total (Ibnelbachyr et al., in preparation). In this study, we applied a population genomic framework using WGS data to (i) describe neutral genomic diversity and population structure in the main Moroccan indigenous goat populations (ii) identify potential genomic regions differentially selected among the main populations according to their specific traits. To address these issues, we sequenced at 12× coverage 44 goats representing the Moroccan-wide geographic diversity of the three main goat indigenous populations in the country.

## Material and methods

### Sampling

**Figure 1:**
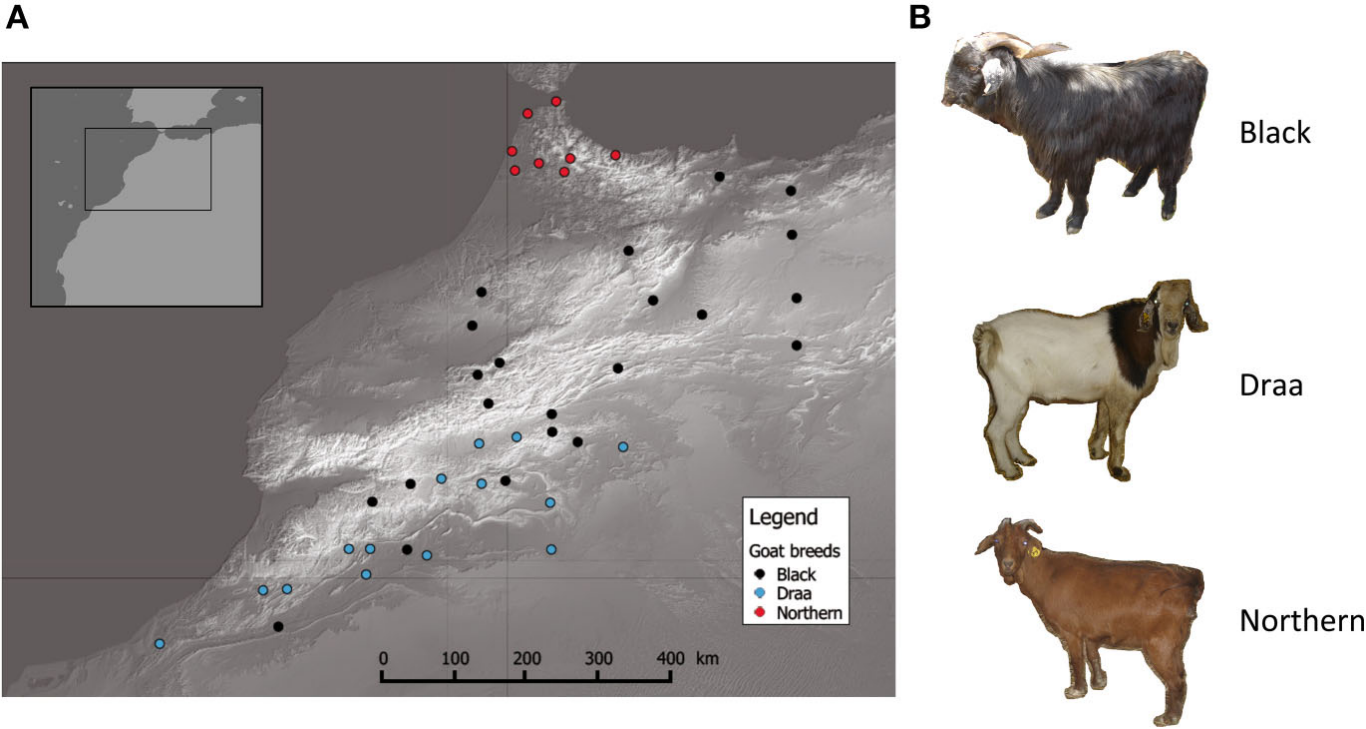
**Distribution of goats sampled**. **(A)** Geographic map showing the distribution of the 44 goats sampled in this study. Each point represents one individual and different colors illustrate different populations. **(B)** Striking phenotypic differences between the 3 main goat populations in Morocco.

Sample collection was performed in a wide part of Morocco [~400,000 km^2^; Northern part of Morocco in latitude range (28°−36°)]. A total of 44 individuals unambiguously assigned to one of the three main indigenous populations (i.e., Black, Draa and Northern) were sampled (Table S1) in a way that maximized individuals' spread over the sampling area. This resulted in sampling spatially distant unrelated individuals, ensuring a spatial representativeness of all regions (Figure 1). For each individual, tissue samples were collected from the distal part of the ear and placed in alcohol for 1 day, and then transferred to a silica-gel tube until DNA extraction.

### Production of WGS data

DNA extractions were done using the Puregene Tissue Kit from Qiagen® following the manufacturer's instructions. Then, 500 ng of DNA were sheared to a 150–700 bp range using the Covaris® E210 instrument (Covaris, Inc., USA). Sheared DNA was used for Illumina® library preparation by a semi-automatized protocol. Briefly, end repair, A tailing and Illumina® compatible adaptors (BiooScientific) ligation were performed using the SPRIWorks Library Preparation System and SPRI TE instrument (Beckmann Coulter), according to the manufacturer protocol. A 300–600 bp size selection was applied in order to recover the most of fragments. DNA fragments were amplified by 12 cycles PCR using Platinum Pfx Taq Polymerase Kit (Life® Technologies) and Illumina® adapter-specific primers. Libraries were purified with 0.8× AMPure XP beads (Beckmann Coulter). After library profile analysis by Agilent 2100 Bioanalyzer (Agilent® Technologies, USA) and qPCR quantification, the libraries were sequenced using 100 base-length read chemistry in paired-end flow cell on the Illumina HiSeq2000 (Illumina®, USA).

### WGS data processing

Paired-end reads were mapped to the goat reference genome (CHIR v1.0, GenBank assembly GCA_000317765.1) (Dong et al., 2013) using BWA mem (Li and Durbin, 2009). The BAM files produced were then sorted using Picard SortSam and improved using Picard MarkDuplicates (http://picard.sourceforge.net), GATK RealignerTargetCreator, GATK IndelRealigner (Depristo et al., 2011), and Samtools calmd (Li et al., 2009). Variant calling was done using three different algorithms: Samtools mpileup (Li et al., 2009), GATK UnifiedGenotyper (McKenna et al., 2010), and Freebayes (Garrison and Marth, 2012).

There were two successive rounds of filtering variant sites. Filtering stage 1 merged together calls from the three algorithms, whilst filtering out the lowest-confidence calls. A variant site passed if it was called by at least two different calling algorithms with variant phred-scaled quality >30. An alternate allele at a site passed if it was called by any one of the calling algorithms, and the genotype count >0. Filtering stage 2 used Variant Quality Score Recalibration by GATK. First, we generated a training set of the highest-confidence variant sites where (i) the site is called by all three variant callers with variant phred-scaled quality >100, (ii) the site is biallelic (iii) the minor allele count is at least 3 while counting only samples with genotype phred-scaled quality >30. The training set was used to build a Gaussian model using the tool GATK VariantRecalibrator using the following variant annotations from UnifiedGenotyper: QD, HaplotypeScore, MQRankSum, ReadPosRankSum, FS, DP, InbreedingCoefficient. The Gaussian model was applied to the full data set, generating a VQSLOD (log odds ratio of being a true variant). Sites were filtered out if VQSLOD < cutoff value. The cutoff value was set for each population by the following: Minimum VQSLOD = {the median value of VQSLOD for training set variants} − 3^*^ {the median absolute deviation VQSLOD of training set variants}. Measures of the transition / transversion ratio of SNPs suggest that this chosen cutoff criterion gives the best balance between selectivity and sensitivity. Genotypes were improved and phased by Beagle 4 (Browning and Browning, 2013), and then filtered out where the genotype probability calculated by Beagle is less than 0.95.

The whole mitochondrial genome (mtDNA) was assembled from a subset of random 20,000,000 reads using the ORGASM tool (Coissac, unpublished). We then extracted the sequence of the HVI segment of the control region for each individual in order to compare with the haplogroup references discovered worldwide (see below).

### Population genomic analyses

#### Characterisation of mtDNA diversity

The number of polymorphic sites and the number of haplotypes were calculated from the whole mitochondrial sequences using DNAsp (Librado and Rozas, 2009). We also calculated these parameters for the hyper variable segment (HVI) of the control region, for which 22 reference sequences representing the diversity of the 6 haplogroups found over the world were available (Naderi et al., 2007). We were interested in the level of resolution of the HVI segment to discriminate the different haplotypes compared to the whole mitochondrion.

Then, using the sequences corresponding to the HVI segment for our dataset and the reference sequences, we drew a network of the haplotypes to identify the different haplogroups present in our dataset. The best evolutionary model was determined using jModelTest v 2.1.4 (Darriba et al., 2012). A median joining network representing the relationships between haplotypes was drawn using SplitsTree4 (Huson and Bryant, 2006).

#### Characterisation of neutral nuclear diversity

Neutral nuclear genomic variations were characterized to evaluate the level of genetic diversity present in Morocco and within populations. The total number of variants and the number of variants within each population were calculated. Allele frequencies and the percentage of exclusive variants (i.e., variants polymorphic in only one population) were estimated at the population scale using the Perl module vcf-compare of Vcftools (Danecek et al., 2011). The level of nucleotide diversity (π) was calculated within each population and averaged over all of the biallelic and fully diploid variants for which all individuals had a called genotype. The observed percentage of heterozygote genotypes per individual (*Ho*) was calculated considering only the biallelic SNPs with no missing genotype calls. From *Ho*, the inbreeding coefficients (*F*) were calculated for each individual using population allelic frequencies over all 44 individuals. The relatedness among individuals was assessed using the pairwise identity-by-state (*IBS*) distances calculated as the average proportion of alleles shared using Vcftools.

Pairwise linkage disequilibrium (LD) was assessed through the correlation coefficient (*r*^2^). It was estimated in 5 segments of 2 Mb on different chromosomes (physical positions between 5 and 7 Mb on chromosomes 6, 11, 16, 21, and 26). LD was estimated either by using the whole set of reliable variants or after discarding rare variants with a minor allele frequency (MAF) less than 0.05. For both estimations, we calculated *r*^2^ values between all pairs of bi-allelic variants (SNPs and indels) on the same segment using Vcftools. Inter-SNP distances (kb) were binned into the following 7 classes: 0–0.2, 0.2–1, 1–2, 2–10, 10–30, 30–60, and 60–120 kb and observed pairwise *LD* was averaged for each inter-SNP distance class and used to draw *LD* decay. Due to the insufficient number of individuals per population we made these estimations for the whole set of individuals without considering each population individually.

Genetic structure was assessed using three different methods: (i) a principal component analysis (PCA) was done using an *LD* pruned subset of bi-allelic SNPs. *LD* between SNPs in windows containing 50 markers was calculated before removing one SNP from each pair where *LD* exceeded 0.95. Subsequently, only 12,543,534 SNPs among a total of 22,304,702 bi-allelic SNPs were kept for this analysis. The R package adegenet v1.3-1 (Jombart and Ahmed, 2011) was used to run PCA and Plink v1.90a (https://www.cog-genomics.org/plink2) was used for *LD* pruning. (ii) An analysis with the clustering method sNMF (Frichot et al., 2014) was carried-out. This method was specifically developed to analyse large genomic datasets in a fast, efficient and reliable way. It is based on sparse non-negative matrix factorization to estimate admixture coefficients of individuals. All biallelic variants were used and five runs for each *K* value from 1 to 10 were performed using a value of *alpha* parameter of 8. For each run, the cross-entropy criterion was calculated with 5% missing data to identify the most likely number of clusters. The run showing the lowest cross-entropy value for a given K was considered. (iii) Finally, the *Fst* index was estimated according to Weir and Cockerham (1984) for each polymorphic site and then weighted to obtain one value over the whole genome. The overall *Fst* between the three groups and the population pairwise values were calculated using Vcftools.

#### Detection of selection signatures

A genome scan approach was performed using the XP-CLR method (Chen et al., 2010) to identify potential regions differentially selected among the three populations. It is a likelihood method for detecting selective sweeps that involves jointly modeling the multi-locus allele frequency differentiation between two populations. This method is robust to detect selective sweeps and especially with regards to the uncertainty in the estimation of local recombination rate (Chen et al., 2010). Due to the absence of genomic position, the physical position (1 Mb ≈ 1 cM) was used. An in-house script based on overlapped segments of a maximum of 27 cM was designed to estimate and assemble XP-CLR scores using the whole set of bi-allelic variants. Overlapping regions of 2 cM were applied and the scores related to the extreme 1 cM were discarded, except at the starting and the end of chromosomes on the CHIR v1.0 assembly. XP-CLR scores were calculated using grid points spaced by 2500 bp with a maximum of 250 variants in a window of 0.5 cM and by down-weighting contributions of highly correlated variants (*r*^2^ > 0.95) in the reference group.

To equilibrate the number of individuals per population, only 14 Black goats were randomly sampled among the 22. They were included with the 14 Draa and the 8 Northern individuals. Each population was tested using a reference group including individuals from the two other populations. The 0.1% genomic regions with highest XP-CLR scores revealed by the analysis were identified and lists of genes partially or fully covered by these regions were then established. To ensure the coverage of short genes (i.e., genes shorter than the distance between adjacent grid points), two segments of 1500 bp each surrounding both sides of genes were also considered. NCBI databases were used to identify coordinates of the 20700 annotated autosomal genes on the CHIR v1.0 genome assembly (http://www.ncbi.nlm.nih.gov/genome?term=capra%20hircus).

#### Gene Ontology enrichment analyses

To explore the biological processes in which the top candidate genes are involved, Gene Ontology (GO) enrichment analyses were performed using the application GOrilla (Eden et al., 2009). The 12,669 goat genes associated with a GO term were used as background reference. Significance for each individual GO-identifier was assessed with *P*-values that were corrected using FDR *q*-value according to the Benjamini and Hochberg (1995) method. GO terms identified in each population were clustered into homogenous groups using REVIGO (Supek et al., 2011). Medium similarity among GO terms in a group was applied and the weight of each GO term was assessed by its *p*-value.

## Results

### Phylogeny of mtDNA genomes

**Figure 2:**
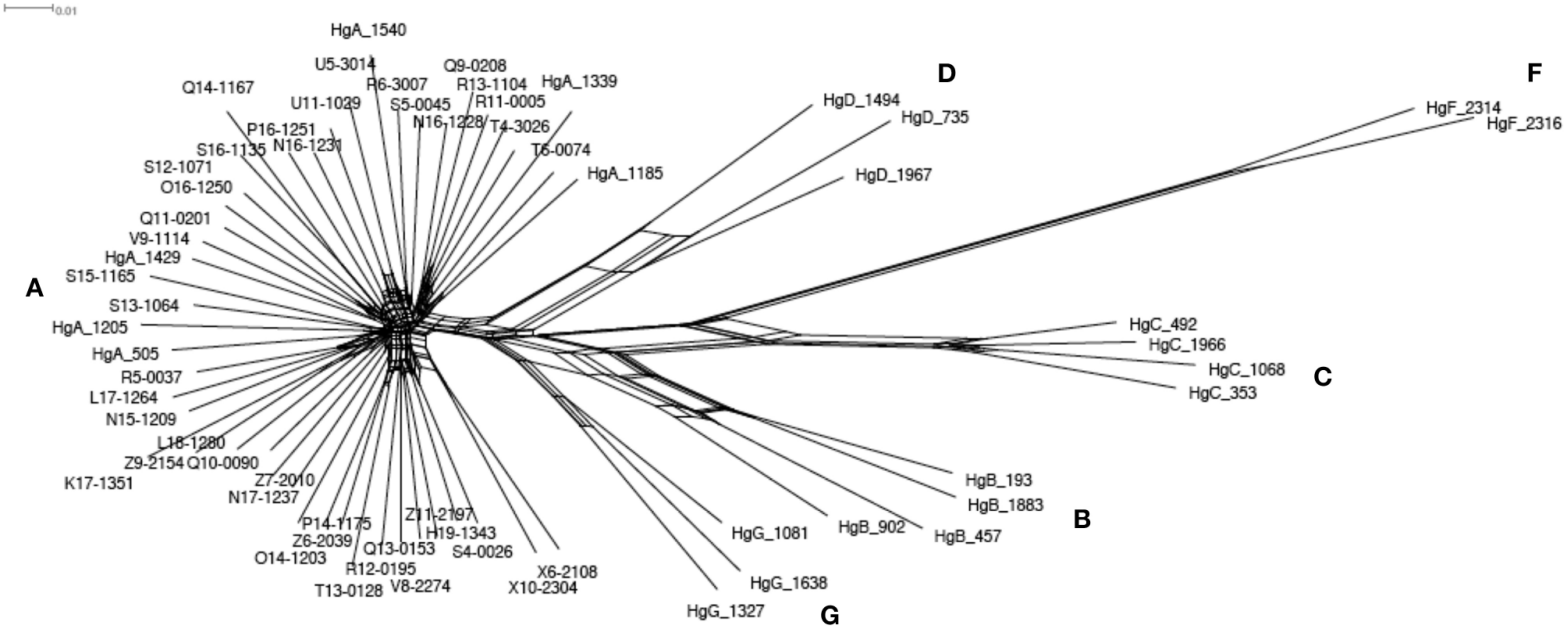
**Phylogenetic network based on the mitochondrial HVI segment of the control region**. Sequences of 41 Moroccan goats and the 22 references representing the worldwide diversity (Naderi et al., 2007) were used. The 22 reference identifiers start with ≪ Hg ≫ and the following letter indicate which haplogroup each belongs to. The other identifiers correspond to the Moroccan goats. The red letters give the names of the 6 haplogroups.

The whole mitochondrial genome was assembled successfully for 41 individuals and represented 16,651 bp length sequences. A total of 239 polymorphic sites were detected, which allowed discriminating 41 haplotypes. In an alternative complementary approach, the 481 bp length sequenced of the HVI segment of the control region was extracted, and this revealed 64 polymorphic sites identifying 40 single haplotypes. We constructed a network using the GTK + G + I model, which showed the best likelihood. The network (Figure 2) including the 22 reference haplotypes (i.e., haplogroups A, B, C, D, F, and G; Naderi et al., 2007) showed that the 40 haplotypes all belonged to the haplogroup A. We did not detect any coherent pattern of geographic structure among the haplotypes. There was also no clear differentiation of the haplotypes according to the three considered populations.

### Neutral diversity from WGS data

The whole nuclear genomes were assembled on the goat reference genome CHIR1.0 along the 30 chromosomes. We mapped unambiguously 99.0% (±0.1%) of reads to the CHIR v1.0 assembly. However, the mapped reads properly paired constituted 90.3% (±0.1%) of reads in average. After the filtering processes, a total of 24,022,850 variants were found to be polymorphic in the total dataset among which 22,396,750 were SNPs and 1,626,100 were small indels. There were a total of 15,948,529 transitions and 6,540,478 transversions leading to a ts/tv ratio of 2.44. Due to differences in quality among individuals, the number of variants called per individual was at least 23,273,239 and 24,003,837 on average. As a consequence, a total of 23,059,968 variants showed no missing genotype over the 44 samples, among which 22,963,257 were biallelic.

**Figure 3:**
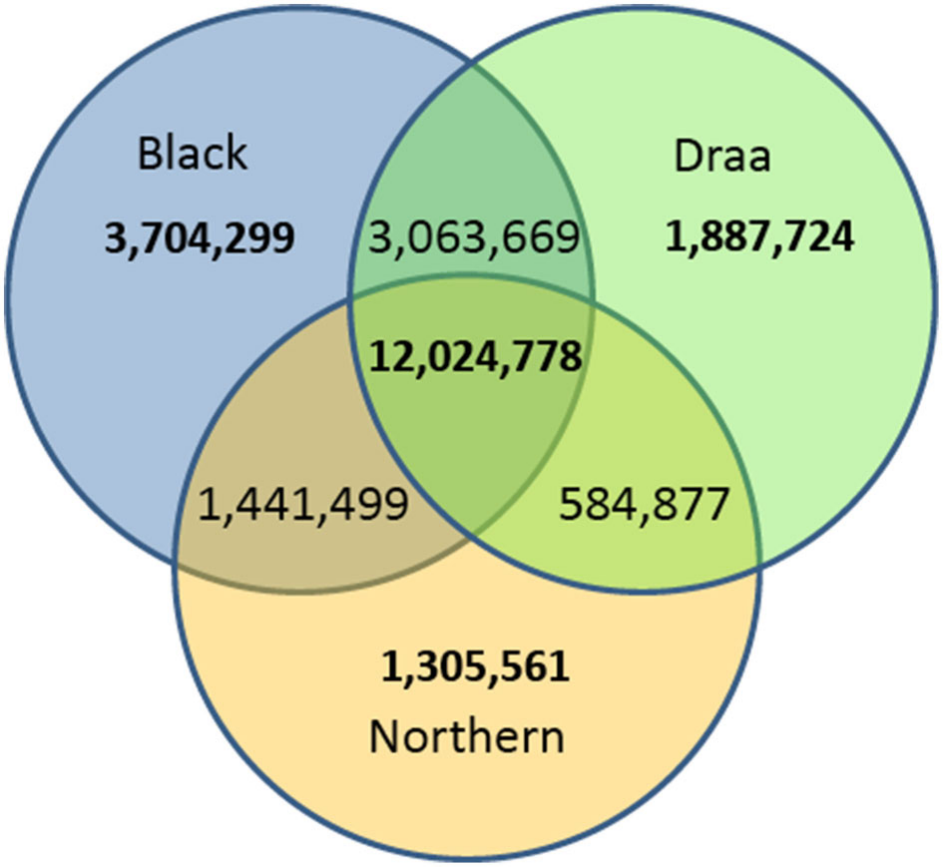
**Venn diagram of the number of polymorphic variants in the three Moroccan goat populations**.

Among the 24,022,850 polymorphic variants, only 12,024,778 variants were polymorphic within each of the three populations. The remaining variants were either polymorphic in only one or in two populations. When considering variants exclusive to each population, 3,704,299 were found polymorphic only in the Black population (*n* = 22), 1,887,724 only in the Draa population (*n* = 14) and 1,305,561 only in the Northern population (*n* = 8) (Figure 3). Rare variants (MAF < 0.05) represented a total of 10,892,203 (45.3%).

Considering the 44 goats together, the average nucleotide diversity (π) calculated from 22,963,257 biallelic variants without missing genotype calls was 0.180. The Draa and the Black populations displayed similar π values amounting to 0.180 and 0.181 respectively. Among the 8 individuals representing the Northern population, π was slightly higher, amounting to 0.189. The observed percentage of heterozygote genotypes per individual (*Ho*) was 17.2% on average, ranging from 12.1% to 18.4%. The average inbreeding coefficient (F) was globally rather low (0.05 ± 0.07) and values were evenly distributed among populations. Similar average values were obtained for the Northern and Black populations (respectively 0.04 ± 0.07 and 0.04 ± 0.05). The Draa goats were slightly more inbred (average *F* = 0.07 ± 0.09), particularly due to one individual showing *F* = 0.32.

**Figure 4:**
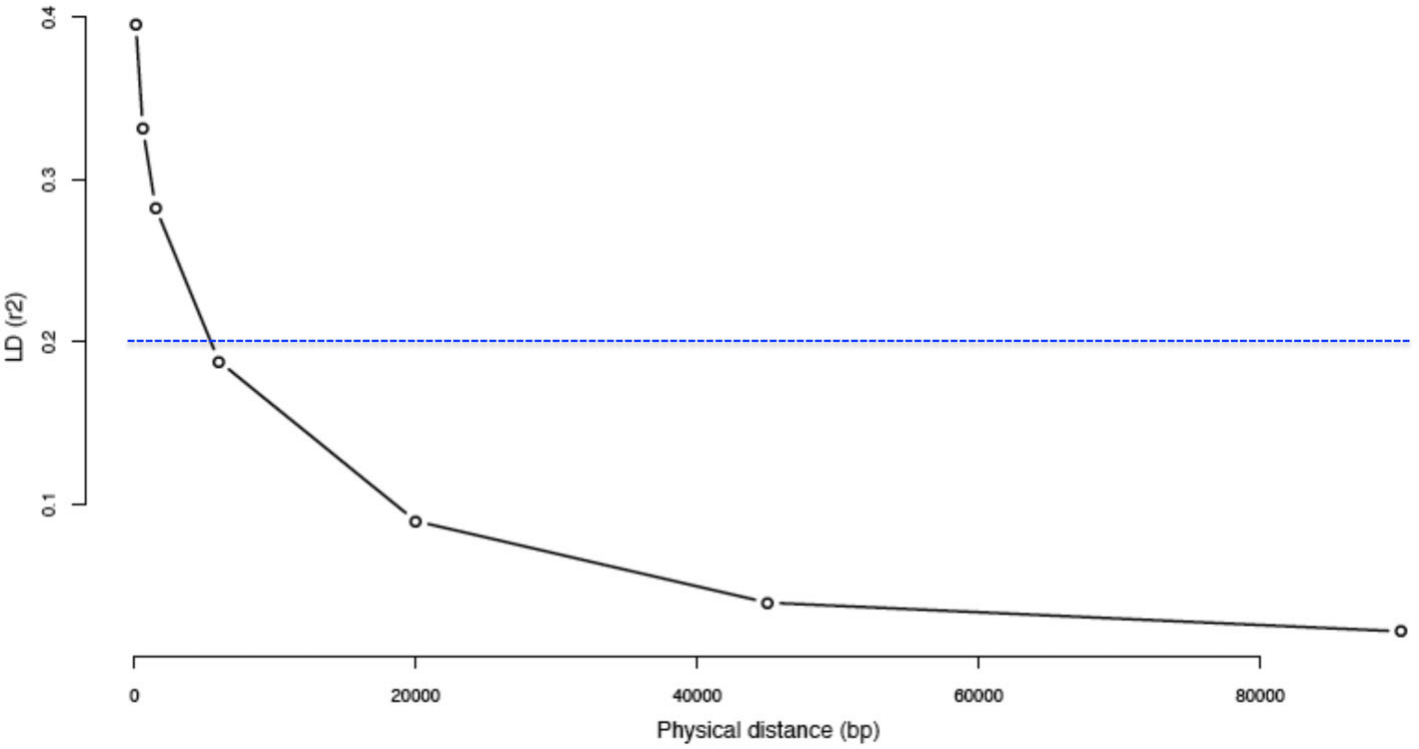
**Decay of linkage disequilibrium (*r*^2^) as a function of physical distance by excluding “rare” variants**. The Linkage Disequilibrium (*LD*) was calculated for the 44 Moroccan goats on 5 different segments of 2 Mb each on 5 different chromosomes. Inter-variant distances (bp) were binned and averaged into the classes: 0–0.2, 0.2–1, 1–2, 2–10, 10–30, 30–60, and 60–120 kb.

We assessed LD by calculating the pairwise *r*^2^ values between polymorphic sites for five chromosome regions. When withdrawing rare variants (i.e., MAF < 0.05), the average *r^2^* value was 0.40 for the first bin (0–0.2 kb) and decayed to less than 0.20 in 5.4 kb (Figure 4). Using the whole set of reliable variants, the average *r*^2^ was 0.21 for the first bin and decreased rapidly to less than 0.20 in 239 bp of distance. Moreover, it decayed to less than 0.15 in about 1.33 kb distance (Figure S2).

Among the three populations, the level of genetic differentiation over the whole nuclear genome was extremely low (*Fst* = 0.0024). The pairwise *Fst* values varied from 0.001 for the Black-Draa comparison to 0.004 for the Northern-Draa comparison. Between the Black and Northern populations the pairwise *Fst* was 0.003.

The PCA analysis showed a very low population structure in the 44 Moroccan goats. The 3 main principal components (PCs) explained 5.8% of variance. The first PC tended to distinguish the Northern and Draa populations while the Black populations formed an in-between group. The second PC acted predominantly to distinguish individuals within the Draa and the Northern populations (Figure S1).

**Figure 5:**
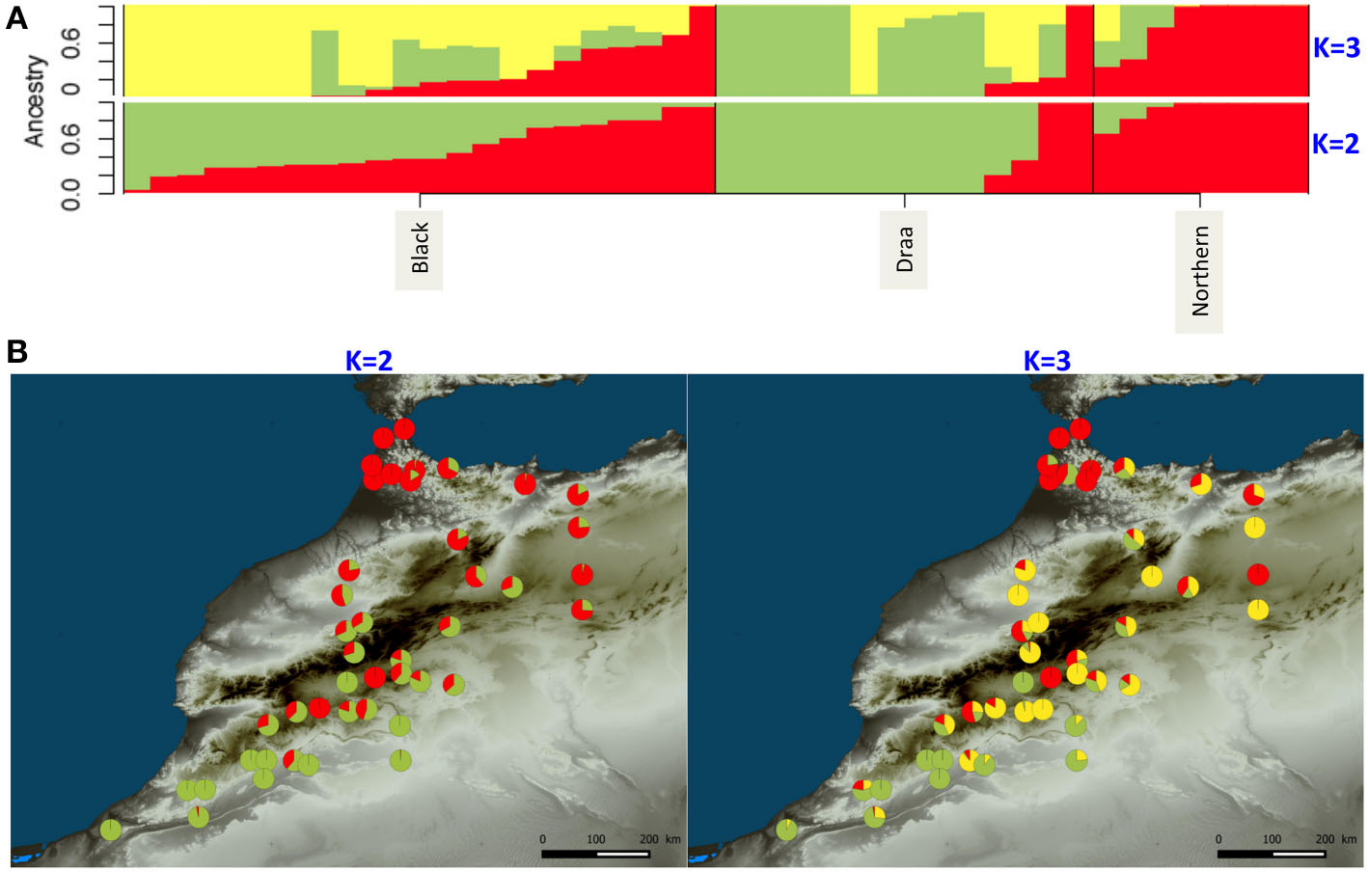
**WGS ancestry estimates for Moroccan goats for *K* = 2 and *K* = 3 clusters using sNMF (Frichot et al., 2014)**. **(A)** Each bar represents one individual. Different colors illustrate the assignment proportion (Q score) to each one of the assumed clusters. **(B)** Geographical distribution of individual *Q*-score values.

The clustering analysis of the genetic structure using sNMF (Frichot et al., 2014) showed that the 44 Moroccan goats belonging to the three populations were more likely represented by only one cluster according to the “crossentropy” criterion (lower values for *K* = 1). However, this criterion is not straightforward and when increasing until *K* = 3 we observed a weak pattern of genetic structure (Figure 5). At *K* = 2, the Northern goats were all strongly assigned to one distinct cluster. The second cluster was characterized by high assignment from the Draa population, except for two individuals that belong to the same cluster as the Northern goats. Finally, the Black goats showed variable levels of admixture between the two clusters (Figure 5A). When mapping the assignment results on a map we observed a geographic pattern with one cluster represented mainly in the north of Morocco (red component; Figure 5B) and the second cluster more present in the south (Figure 5B). At *K* = 3, the additional cluster was mostly represented in the Black goats which are located in the center of the sampling area (Figure 5A). The two other clusters still mostly represented the separation of Northern and Draa populations but the pattern was less evident. It was difficult to disentangle the relationship of genetic structure with populations and geography because the two factors were confounding.

### Selection signatures

**Figure 6:**
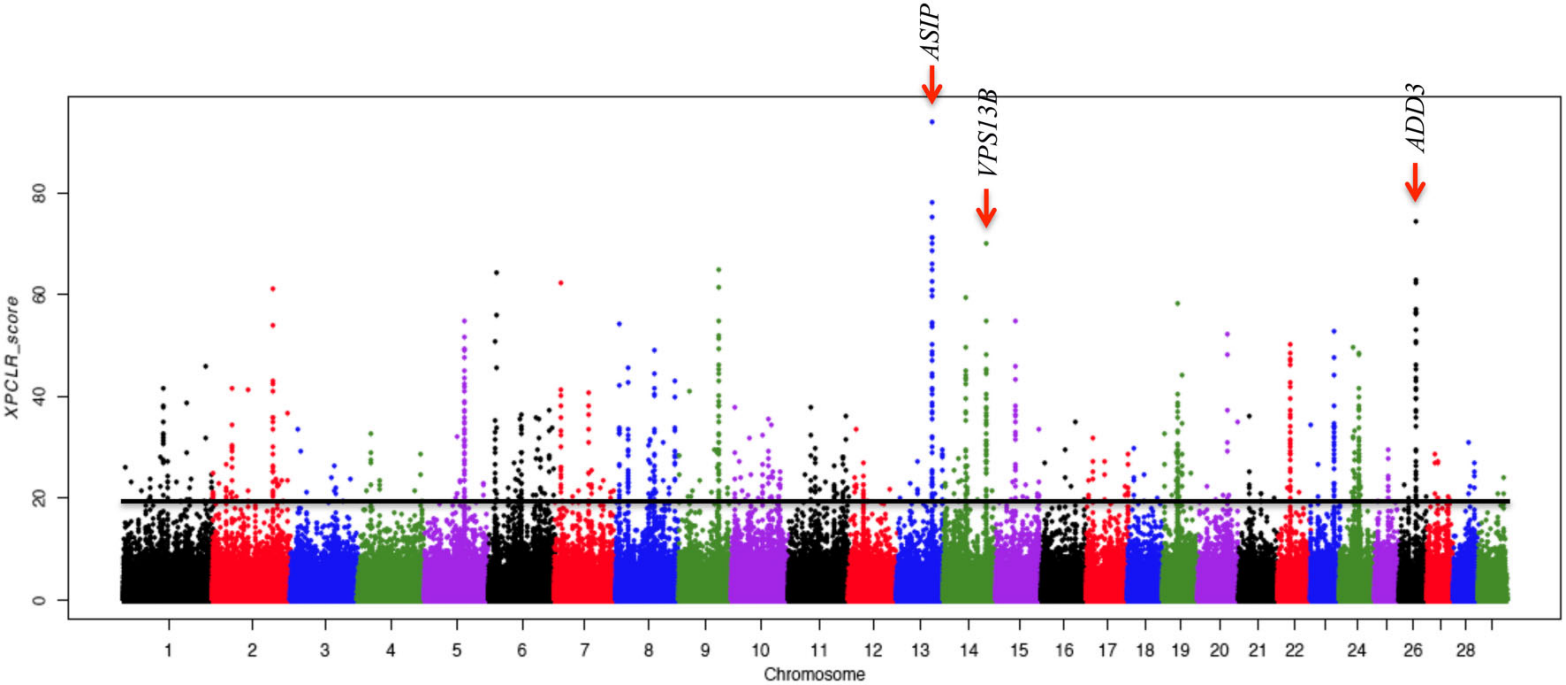
**Plot of XP-CLR scores along autosomes in selective sweep analysis for the Draa goat population**. The horizontal line indicates a 0.1% autosomal-wide cut-off level. Red arrows and names indicate the top three candidate genes.

We applied the XP-CLR genome scan method (Chen et al., 2010) on the whole genomes of 36 goats from the three phenotypic populations (14 Black, 14 Draa, and 8 Northern). We identified selective sweep genes in each population considering the top 0.1% genome-wide scores. Our approach highlighted respectively 142, 167, and 176 candidate genes in the Black, Draa, and Northern populations. The region showing the strongest XP-CLR score was located on chromosome 6 for the Black goats (Figure S3) and on chromosome 22 for the Northern goats (Figure S4), but they did not match any annotated gene. The annotated genes showing the strongest selective sweeps were *HTT, MSANTD1*, and *LOC102170765* in the Black goats, and *FOXP2, TRAP1* and *DNASE1* in the Northern goats (Table 1). In the Draa population, the highest XP-CLR scores corresponded to *LOC102190531, ADD3*, and *ASIP* genes (Figure 6). The enrichment categories of the identified candidate genes in the Black goats were associated with 15 GO terms (Table S2). They clustered into the following four differentiated categories by REVIGO (Supek et al., 2011): tube development, calcium ion transmembrane import into mitochondrion, negative regulation of transcription from RNA polymerase II promoter during mitosis and response to fatty acid. The enrichment of the identified candidate genes in Draa goats highlighted the significance of 25 GO terms (Table S3) clustering into five differentiated categories: regulation of respiratory gaseous exchange, behavior, postsynaptic membrane organization, protein localization to synapse, and neuron cell-cell adhesion. In the Northern goats, we did not find significant enrichment categories for the candidate genes identified.

**Table 1:** **Top-20 candidate genes under positive selection in each Moroccan goat population using the top-0.1% XP-CLR scores autosomal-wide cut-off level**.

**Black population**					**Draa**					**Northern population**				
**Gene**	**Chr**	**Number of top-scores**	**Distance/grid point**	**Higher score**	**Gene**	**Chr**	**Number of top-scores**	**Distance/grid point**	**Higher score**	**Gene**	**Chr**	**Number of top-scores**	**Distance/grid point**	**Higher score**
*HTT*	6	29	4739	82.6	*LOC102190531*	13	9	2493	94.0	*FOXP2*	4	14	33163	48.7
*MSANTD1*	6	3	5501	61.4	*ADD3*	26	24	5409	74.4	*TRAP1*	25	5	3977	42.8
*LOC102170765*	6	1	699	54.0	*ASIP*	13	2	995	71.3	*DNASE1*	25	4	2485	41.8
*FAM160B1*	26	2	42409	45.6	*VPS13B*	14	36	21697	70.1	*FAM227B*	10	9	25094	39.8
*STRIP1*	3	5	3069	43.6	*RALY*	13	9	5294	66.1	*CREBBP*	25	14	9497	38.6
*NDUFA6*	5	4	2786	41.8	*ICAM3*	7	5	1696	62.4	*PAPSS2*	26	1	43841	35.7
*HNRNPA3*	2	3	1183	40.3	*HIVEP2*	9	15	6353	61.4	*SLX4*	25	2	9472	32.4
*KITLG*	5	7	15223	39.7	*GGH*	14	10	2984	59.3	*PGM5*	8	9	23504	32.0
*ALX3*	3	1	7499	39.6	*PLSCR3*	19	2	1770	58.2	*BCAS3*	19	11	53928	31.4
*IFT88*	12	13	4139	39.6	*SOX6*	15	17	28387	54.8	*GALK2*	10	3	51372	31.2
*XPO4*	12	25	3141	39.5	*JARID2*	23	27	8506	52.9	*MAB21L1*	12	2	1213	31.0
*VPS13B*	14	16	48818	38.0	*NOL4*	24	6	78026	49.7	*NBEA*	12	31	21437	30.9
*LOC102183160*	14	1	298	37.5	*TIMP3*	5	3	4339	49.4	*LOC102182654*	25	1	2127	30.8
*FLI1*	29	6	10103	36.9	*EIF2S2*	13	2	7340	48.8	*LCOR*	26	7	8873	30.6
*C4H7orf10*	4	11	70095	35.7	*TTC39C*	24	10	10009	48.2	*RANBP10*	18	10	5817	29.5
*TTC21A*	22	3	11417	35.6	*PCBP3*	1	2	103547	46.0	*SLC12A4*	18	2	10049	28.0
*LATS2*	12	4	6149	34.3	*TTPA*	14	7	8967	45.0	*ROR1*	3	5	39379	27.8
*NSMCE2*	14	5	46323	33.7	*ASTN2*	8	11	79003	43.1	*MRPL54*	7	1	2005	27.7
*ATG2B*	21	3	25060	33.4	*GALNT7*	8	10	10949	42.2	*PDE1A*	2	2	146650	27.4
*FAT2*	7	2	42599	33.4	*MUC13*	1	7	3341	41.7	*KRT8*	5	2	3717	27.3

*Coordinates of 20700 autosomal genes on the CHIR v1.0 goat assembly were used to identify candidate genes matching XP-CLR top scores. Genes were ranked according to the higher XP-CLR score. Chr, Chromosome. Number of top-scores, Number of grid points among the top-0.1% XP-CLR scores matching the gene. Distance/grid point: gene length/number of top-scores. Grid points in XP-CLR analysis were separated by 2.5 Kb*.

## Discussion

Indigenous/traditional goats have been raised for a long time for various purposes and they have gradually accumulated several traits making them well adapted to their environments. The mechanisms underlying these adaptive traits have been poorly studied until now. The recent development of sequencing technologies has now made possible the sequencing of individuals' whole genomes and this may greatly expand our understanding of genomic diversity. Except for a few studies based on medium density SNP panels (about 50,000 SNPs) (Kijas et al., 2013; Tosser-Klopp et al., 2014), previous population genetic studies on goats have been limited to just a few dozens of markers (i.e., microsatellites). In this study we used variants spanning the whole genome to characterize indigenous goat populations of Morocco.

### Mitochondrial variation

Complete mitochondrial sequences were successfully assembled from a low portion of reads for 41 individuals. In terms of its ability to discriminate between the different haplotypes, the 481 bp length of the HVI segment of the control region was almost as accurate as the whole mitochondrion sequence of 16,651 bp length from which it was extracted. Only a small difference in the total number of haplotypes defined was found (41 against 40 haplotypes respectively). This result shows that despite a low number of variable sites, the dense variability found in the control region (26.8% of the total number of variants for only 2.8% of the sequence length) concentrated most of the phylogenetic information. Thus, the HVI segment of the control region seems to be a good surrogate of the whole mitochondrial polymorphism. This study confirmed previous results based on the HVI segment of the control region (Pereira et al., 2009; Benjelloun et al., 2011) where Moroccan domestic goats showed only haplotypes from the A haplogroup (HgA). In a larger study using 2430 samples with a worldwide distribution, Naderi et al. (2007) found that most of the domestic goats displayed HgA (about 94%). Thus, it seems that the mitochondrial categorization in Morocco is rather representative of the rest of the world, even if the remaining haplogroups were not identified in our sampling. Besides this, the mtDNA diversity was weakly structured according to geography, as already reported by (Benjelloun et al., 2011) on the HVI region.

We did not find any clear structure of the mitochondrial haplotypes among the three populations. The high mitochondrial diversity characterizing these three populations probably indicates the diversity present in the first domesticated goats that arrived in Morocco and/or recurrent gene flows from diverse origins. According to (Pereira et al., 2009), Moroccan goat populations would have been established via two main colonization routes, one a North African land route and the other a Mediterranean maritime route across the Strait of Gibraltar. The high gene flows between populations, mediated by humans, would be ultimately responsible for the absence of structure across Morocco.

### Nuclear neutral variation

Although the low percentage of the properly paired mapped reads (about 10%) in comparison with the percentage of mapped reads (about 99%) would illustrate a possible fragmentation of the genome assembly used, we identified many high confidence variants (approximately 24 million among which 6.8% were small indels) over the whole nuclear genomes of the 44 Moroccan goats studied. This is much higher than was found in all previous studies detecting variants in large sample cohorts from whole genome sequencing. For example, the human 1000 Genomes Project (Altshuler et al., 2012) detected approximately 15 million SNPs and 1 million short indels, while in the 1001 Genomes Project of *Arabidopsis thaliana* about 5 million SNPs and 81,000 small indels were found (Cao et al., 2011). The polymorphism detected in the Moroccan goats remains huge even when considered in proportion to the genome size of the species.

This huge number of variants did not show a strong genetic structure either among populations or over geographic space. The globally weak genetic structure suggests that extensive gene flows along with low level of selection have produced this pattern. Our findings contrast with most previous studies, which generally show a clear structure among goat breeds or populations (Cañon et al., 2006; Agha et al., 2008; Serrano et al., 2009; Di et al., 2011; Hassen et al., 2012; Kijas et al., 2013). Several reasons could explain this difference. First, most of the previous studies used microsatellite markers exhibiting high mutation rate. Thus, compared to SNP markers, microsatellites could more likely show imprints of recent demographic events such as differentiation between recently isolated populations. Moreover, the microsatellite markers generally used (Serrano et al., 2009; Di et al., 2011) were recommended by FAO and designed to exacerbate genetic differentiation among breeds, which was thus artificially inflated. In a more recent study, (Kijas et al., 2013) used a panel of SNP markers from a chip designed with animals representing industrial breeds for the SNP discovery (Tosser-Klopp et al., 2014). In that case the results were certainly inflated by the ascertainment bias due to the chip design. However, it is also likely that in our case the demographic history of Moroccan goats differs from that of the breeds previously studied, and in particular from the ones compared at larger geographic scales such as Europe and Middle East (Cañon et al., 2006), or China, Iran and Africa (Di et al., 2011). The structured diversity found in these latter two studies would result from the strong isolation between countries. However, even at smaller scales the selection pressures exerted by breeding processes and husbandry practices may have increased isolation among breeds, and thus reinforced population differentiation compared to Morocco. The situation found in Morocco is close to the one described by Hassen et al. (2012) for six Ethiopian goat ecotypes, where even with microsatellite markers most of the diversity was found within populations, showing low levels of genetic differentiation. This result was explained by the existence of uncontrolled breeding strategies and agricultural extensive systems. In Morocco, it seems that goat populations have experienced moderate levels of selection and that most of the genetic diversity has been preserved during the breeding process which led to the three phenotypic populations. However, a weak genetic pattern was revealed by sNMF, which seems to be partially related to populations as well as geography. When mapping the clustering results (for *K* = 3, Figure 4B), a pattern appeared across Morocco, with Northern goats displaying a higher assignment probability to one distinct cluster. The Northern population is observably slightly more diverse than the others for which higher numbers of individuals were studied. This higher diversity and the slightly higher genetic differentiation of the Northern goats support the hypothesis of an influence of Iberian gene flows through the strait of Gibraltar in the North of Morocco (Analla and Serradilla, 1997).

The goal of our study was not to visualize the *LD* variations along chromosomes by covering all regions including centromeres and chromosomal inversions that are reportedly characterized by an elevated *LD* (Weetman et al., 2010; Marsden et al., 2014). Rather, we aimed to generate a global representation of *LD* across the genome by covering segments of 2 Mb in 5 different chromosomes taking all the reliable variants found from WGS data. Furthermore, knowing the effect of rare variants on *LD* estimation (Andolfatto and Przeworski, 2001) and to compare our findings with previous studies, we also estimated *LD* after discarding rare variants (MAF < 0.05). The extent of LD reported without rare variants (*r*^2^ < 0.20 after 5.4 kb on average) is clearly shorter compared to all previous studies on farm animals, where it largely exceeds 10 kb for *r*^2^ = 0.20 (Meadows et al., 2008; Villa-Angulo et al., 2009; Wade et al., 2009; McCue et al., 2012; Ai et al., 2013; Veroneze et al., 2013). In these studies, whole genome variants were not available and potential biases due to the use of SNP chips may partially explain the results. However, we consider that our finding would mainly result from the extensive breeding system favoring high gene flows among Moroccan goat populations/herds and low inbreeding and from the absence until now of strong selection during the breeding processes. Results on *LD* and genetic variability illustrate the important diversity present in indigenous populations in comparison with industrial breeds on which previous studies mainly focussed (e.g., Meadows et al., 2008; Villa-Angulo et al., 2009). This should be considered in the establishment of future programs aimed at improving these populations to preserve this highly valuable genetic diversity.

Beside this, when using the whole set of reliable variants we found a much lower *LD* (*r*^20^_0.20_ = 239 bp). We do believe that this value should be considered in genome wide association and genome scan studies. Indeed most of studies remove rare variants for genotyping quality issues. In our case, the quality filtering produced reliable rare variants (about 45%) that would give a more realistic estimation of LD. To our knowledge, very few studies included rare variants to estimate *LD* (e.g., Mackay et al., 2012).

### Selection signatures in moroccan goat populations

The weakly structured genetic diversity in Moroccan goats was suitable to detect selection signatures, avoiding possible false positives potentially generated by genetic structure. Despite a common genomic background and this weak population structure in Moroccan goats, the three main populations have been bred in various conditions and thereby have been subject to different anthropic and environmental selections in their recent history. As a result, they differ in their physiology, behavior and morphology. The observation of rapid phenotypic changes raises the question of the underlying genetic changes that would be shaped by selection. We identified numerous signatures of selection corresponding to genomic regions potentially under selection in each population.

A difficulty in identifying the genes or metabolic pathways under selection resides in the currently incomplete annotation of the goat genome. The stronger selective sweeps corresponded to regions in the Black population (chromosome 6) and in the Northern population (chromosome 22) matching un-annotated genes on the CHIR v1.0 assembly. This is probably due to either the incomplete annotation of the caprine genome or the fact that the selected functional mutations within each of these regions are not located within or close to a protein-coding gene. The incomplete genome annotation prevented us from identifying several known selected genes among Moroccan goat populations. For example, the *melanocortin-1 receptor* (*MC1R*) gene that is reported to be involved in coat color differentiation in goats (e.g., Fontanesi et al., 2009a) is not associated to any chromosome on the CHIR v1.0 assembly. Therefore, we were not able to detect its possible associated signal of selection in populations where the coat color is fixed knowing that we looked for selection signatures on autosomes only. Another problem consisted in the presence of several annotated genes that were not identified (i.e., no known orthologs, gene identifier starting with “LOC”). Thus, many genes potentially under selection could not be used in our GO enrichment analyses (e.g., the higher-score candidate gene in Draa population on Chromosome 13; Table 1). Despite these restrictions, we identified several sets of strong candidate genes in the three studied populations.

In the Black population the top-ranked candidate gene identified was *huntingtin* (*HTT;* Table 1). It has been comprehensively studied in humans where it is associated with Huntington's disease, an inherited autosomal dominant neurodegenerative disorder (Mende-Mueller et al., 2001; Sathasivam et al., 2013). The *HTT* protein directly binds the endoplasmic reticulum (ER) and may play a role in autophagy triggered by ER stress (Atwal and Truant, 2008). Thus, we could speculate a possible involvement of this gene in the adaptation to physiological or pathological conditions leading to ER stress. This gene, among other candidates, was involved in the enrichment of GO terms *pattern specification process* (GO:0007389) and *organ development* (GO:0048513). These two categories were clustered together with the enriched *neuron maturation* term (GO:0042551) (Table S2). Hence, we could hypothesize a possible role of genes involved in these categories in some morphological traits specific to the Black goat population. Besides this, we noticed the enrichment of genes associated with the response to fatty acids GO terms (GO:0070542; GO:0071398). Candidate genes in these categories include *CPT1A* that encodes for a mitochondrial enzyme responsible for the formation of acyl carnitines that enables activated fatty acids to enter the mitochondria (van der Leij et al., 2000; Vaz and Wanders, 2002). The *SREBF1* gene encodes for a family of transcription factors (*SREBPs*) that regulate lipid homeostasis (Yokoyama et al., 1993; Eberle et al., 2004). The *GNPAT* gene encodes an essential enzyme to the synthesis of ether phospholipids. The last gene in these categories is *CPS1* and it encodes for a mitochondrial enzyme that catalyzes synthesis of carbamoyl phosphate (Aoshima et al., 2001). This suggests that selection acted upon the metabolism of fatty acids and lipids in the Black population, reflecting the possible development of an effective metabolism that could be linked to a higher amount of volatile fatty acids generated by the rumen microbial flora (Bergman, 1990).

In the Draa population, which is raised in oasis/desert areas and well adapted to high temperatures (Hossaini-Hilali and Mouslih, 2002), the enrichment of GO terms associated with the regulation of respiratory system and gaseous exchange categories (GO:0002087; GO:0043576; GO:0044065) would reflect the likely use of panting in evaporative heat loss. Goats could use panting as well as sweating for body thermo regulation according to the level of hydration and solar radiation (Dmiel and Robertshaw, 1983; Baker, 1989), and the type of regulatory system also depends on the breed/population (e.g., The Black Bedouin goats of Sinai Peninsula that use sweating in preference to panting) (Dmiel et al., 1979). Panting compared to sweating helps animals to better preserve their blood plasma volume (no losses of salt) and involves cooling of the blood passing the nasal area, which makes it possible to keep brain temperature lower than body temperature (Baker, 1989). Differences between Draa and Black populations in coat color, hair length and head size (larger in Black, Ibnelbachyr et al., in preparation) would support the hypothesis of different mechanisms of adaptation. Black goats would favor sweating and Draa panting as the more beneficial adaptation to warm environments. Mechanisms underlying dissipation should be further studied in these populations to elucidate the adaptive processes involved.

The enrichment of GO terms associated with lactate transport (GO:0015727; GO:0035873) (Table S3) in the Draa population could be linked to the stronger specific energetic demand associated with pregnancy and lactation in this population. The prolificacy in this population is much higher than in the rest of Moroccan goats (about 1.51 kids/birth vs. about 1 kid/birth; Ibnelbachyr et al., 2014). Thereby lactate transport may play a crucial role to meet this higher energetic requirement by shuttling lactate to a variety of sites where it could be oxidized directly, re-converted back to pyruvate or glucose and oxidized again, allowing the process of glycolysis to restart and ATP provision maintained (Brooks, 2000; Philp et al., 2005). This corroborates the higher concentration of lactate in cells during lactation than during dry-off period 5 weeks before parturition in cattle reported by Schwarm et al. (2013). Besides this, a top candidate gene in the Draa population was the *agouti signaling protein* (*ASIP*) (Table 1), which plays a key role in the modulation of hair and skin pigmentation in mammals (Lu et al., 1994; Furumura et al., 1996; Kanetsky et al., 2002) by antagonizing the effect of the *melanocortin-1 receptor gene* (*MC1R*) and promoting the synthesis of phaeomelanin, a yellow–red pigment (Hida et al., 2009). *ASIP* was associated with different coat colors in cattle and sheep (Seo et al., 2007; Norris and Whan, 2008). The strong selective sweep related to this gene could be linked to the higher variation in Draa's coat color when compared to other populations (Ibnelbachyr et al., in preparation). This variation in coat color was highly represented in the 14 Draa samples used in this study (Table S4). However, previous studies focussing on this gene identified an important polymorphism in worldwide goat breeds without any clear association with differences in coat color (Badaoui et al., 2011; Adefenwa et al., 2013). Fontanesi et al. (2009b) reported the presence of a copy number variation (CNV) affecting *ASIP* and *AHCY* genes, and might be associated to the white color in Girgentana and Saneen breeds. Nevertheless, the design of our study was not adapted to identify CNV and we cannot link the selection signature detected here in this gene to the findings of this study.

In the Northern population, no GO term was enriched but the second ranked candidate gene identified was *TRAP1*, which encodes a mitochondrial chaperone protein (Felts et al., 2000). Under stress conditions this gene was shown to protect cells from reactive oxygen species, (ROS)-induced apoptosis and senescence (Im et al., 2007; Pridgeon et al., 2007). Such regulation of the cellular stress response would play a role in the adaptation of this population to harsh environments (e.g., mountainous areas in the North of Morocco).

Finally, several strong signals of selection pointed to genes or pathways for which possible functions remained ambiguous. For example in the Northern population, the strong signal of selection associated with *FOXP2*, which encodes for a regulatory protein, is required for proper development of language in Humans (Lai et al., 2001), song learning in songbirds (Haesler et al., 2004), and learning of rapid movement sequences in mice (Groszer et al., 2008). This gene could be involved in learning but its possible functions in goats cannot be hypothesized easily. A similar case was found in the Draa population for which GO categories linked to behavior and vocalization behavior (GO:0071625; GO:0030534; GO:0007610) were enriched. We were not able to predict the possible functions of these genes. Furthermore, the *NR6A1* gene that was identified potentially under selection in Draa (within the top 0.1% XP-CLR scores) was previously associated with the number of vertebras in pigs (Mikawa et al., 2007; Rubin et al., 2012). Considering the larger body length and size in this population in comparison with the Black population (Ibnelbachyr et al., in preparation), we could hypothesize a similar role of this gene in the body elongation in goats. A future characterization of this morphologic trait in Draa goats would confirm or refute this hypothesis.

## Conclusion

Our study characterized whole genome variation in the main goat indigenous populations at a countrywide scale in an unprecedented way. The whole genome data and the wide geographic spread of animals allowed for a precise characterization of the distribution of genomic diversity in various populations. The position of Morocco has made it subject to various colonization waves for domestic animals. Additionally, previous and present management schemes have favored gene flow between goat populations. This created and maintained a very high level of total genetic diversity that is weakly structured according to geography and populations. A part of the overall diversity corresponded to potentially adaptive variation, as several genes appeared to be under selection. The different populations studied appeared to bear specific adaptations, even when submitted to similar conditions such as those related to a warm/desert context. This would demonstrate the potential of different indigenous livestock populations to constitute complementary reservoirs of possibly adaptive diversity that would be highly valuable in the context of global environmental changes. However, these populations are threatened due to their substitution by more productive cosmopolitan breeds that should not have the potential to become locally adapted to harsh environments. It is thus extremely important to promote the sustainable management of these genetic resources with emphasis on both overall neutral and adaptive diversity. This study has also identified several genes as potentially under selection and further studies are needed to depict the underlying mechanisms.

## Accession numbers

The accession numbers of the 44 samples in the BioSamples archive, the accession numbers of the sequencing data and aligned bam files in the ENA archive are reported in the Table S1. The variant calls and genotype calls used in this paper are archived in the European Variation Archive with accession ID ERZ020631.

## Author contributions

PT, FP, SJ, PF designed the study. PT and FP supervised the study. BB, MB, MI, MC, AB, AC sampled individuals. AA, SE produced whole genome sequences. BB, FJA, IS, FB, EC, SS, KL, MI, LC analyzed the data and interpreted the results. BB, FJA, FP, KL, SJ, IS, AA wrote the Manuscript. All authors revised and accepted the final version of the manuscript.

### Conflict of interest statement

The authors declare that the research was conducted in the absence of any commercial or financial relationships that could be construed as a potential conflict of interest.
