# Supplementary material for: Characterizing neutral genomic diversity and selection signatures in indigenous populations of Moroccan goats (Capra hircus) using WGS data
Source: Front Genet. 2015 Apr 7;6:107. doi: 10.3389/fgene.2015.00107 (PMC4387958; doi:10.3389/fgene.2015.00107)
Supplement: Supplementary file 1 [file DataSheet1.ZIP › Supplemental Data/Figure S2.pdf]

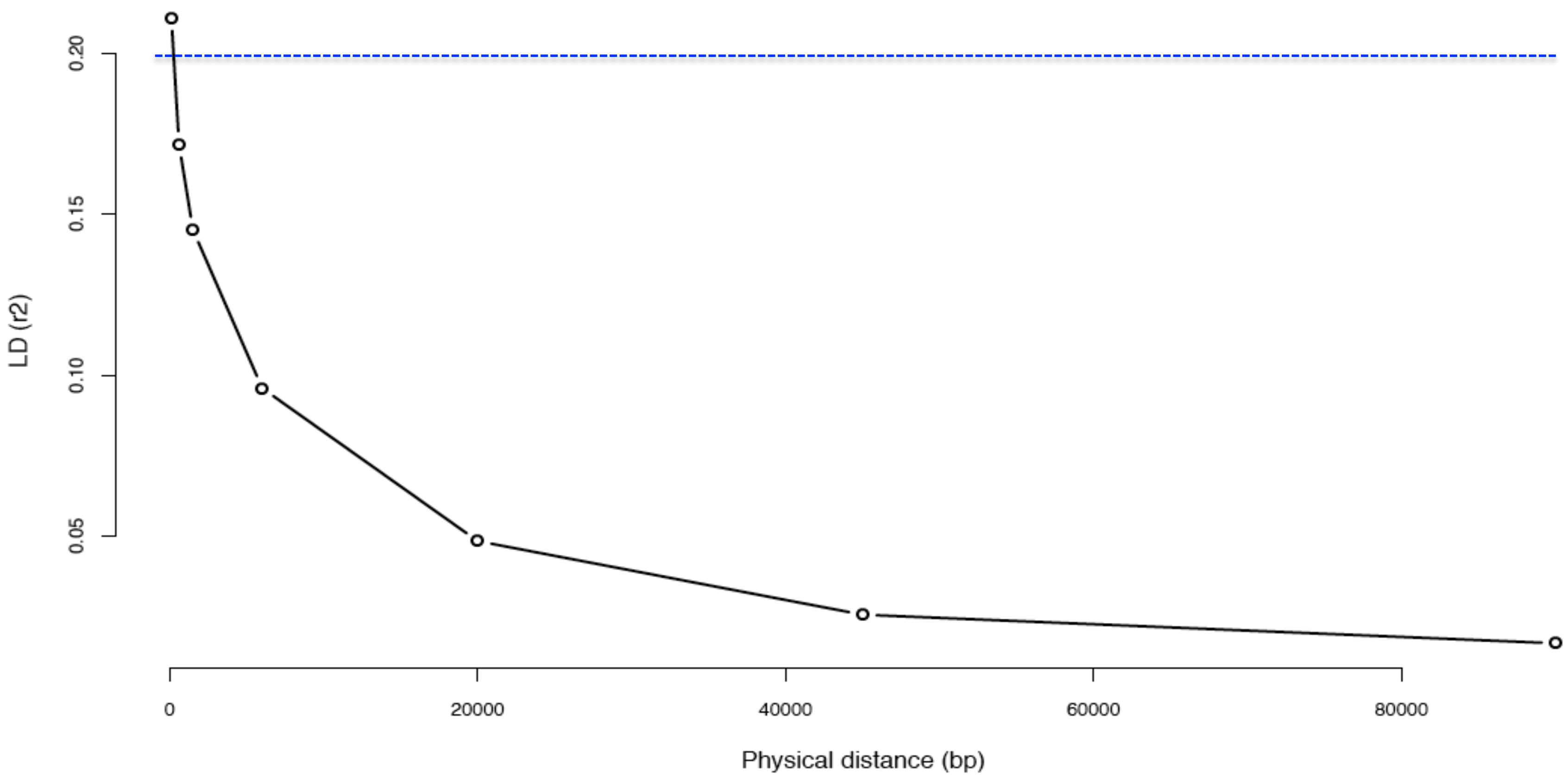

Figure S2: Decay of linkage disequilibrium (r^2) as a function of physical distance including “rare” variants.
The Linkage Disequilibrium (*LD*) was calculated for the 44 Moroccan goats on 5 different segments of 2Mb each on 5 different chromosomes.
Inter-variant distances (bp) were binned and averaged into the classes: 0–0.2, 0.2–1, 1–2, 2–10, 10-30, 30-60 and 60-120 kb.
