# Supplementary material for: Characterizing neutral genomic diversity and selection signatures in indigenous populations of Moroccan goats (Capra hircus) using WGS data
Source: Front Genet. 2015 Apr 7;6:107. doi: 10.3389/fgene.2015.00107 (PMC4387958; doi:10.3389/fgene.2015.00107)
Supplement: Supplementary file 1 [file DataSheet1.ZIP › Supplemental Data/Figure S3.pdf]

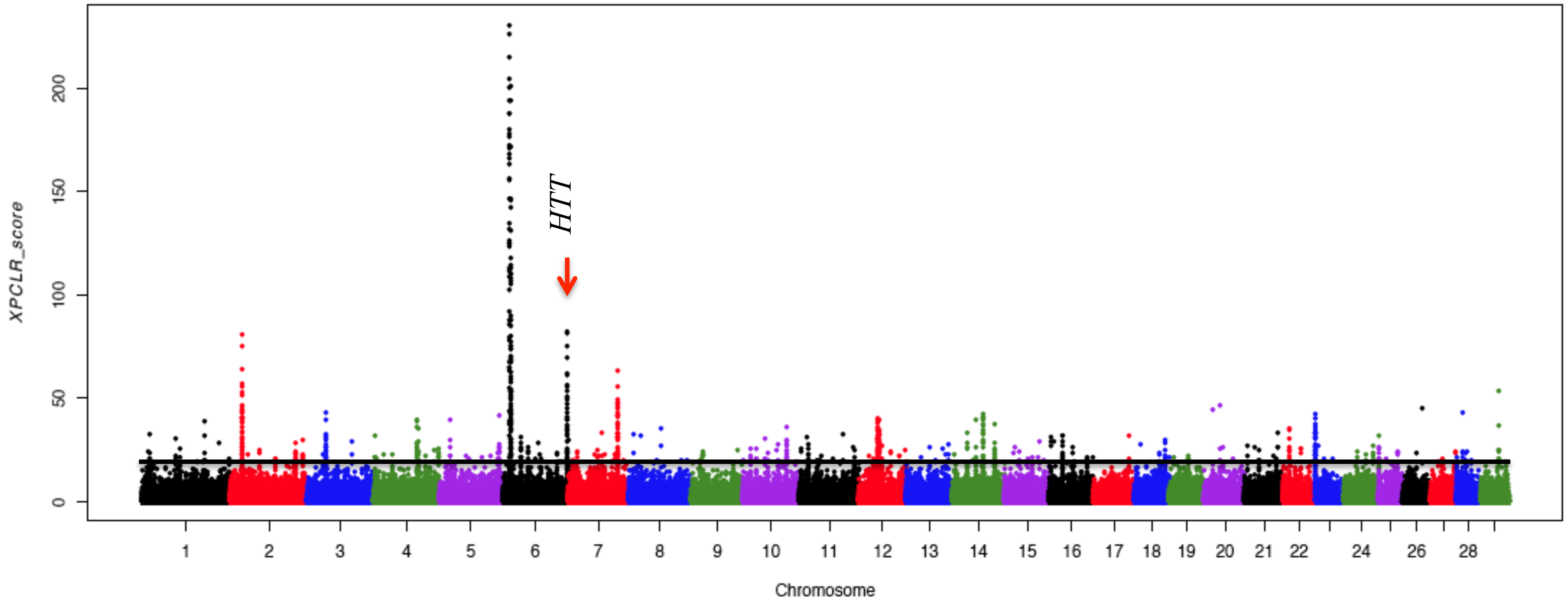

Figure S3: Plot of XP-CLR scores along autosomes in selective sweep analysis for the Black goat population.

The horizontal line indicates a 0.1% autosomal-wide cut-off level. The red arrow and name indicates the top candidate gene. The higher scores linked to the stronger signal on chromosome 6 were not associated to any annotated gene on the goat assembly (CHIR v1.0).
