# Supplementary material for: Characterizing neutral genomic diversity and selection signatures in indigenous populations of Moroccan goats (Capra hircus) using WGS data
Source: Front Genet. 2015 Apr 7;6:107. doi: 10.3389/fgene.2015.00107 (PMC4387958; doi:10.3389/fgene.2015.00107)
Supplement: Supplementary file 1 [file DataSheet1.ZIP › Supplemental Data/Figure S4.pdf]

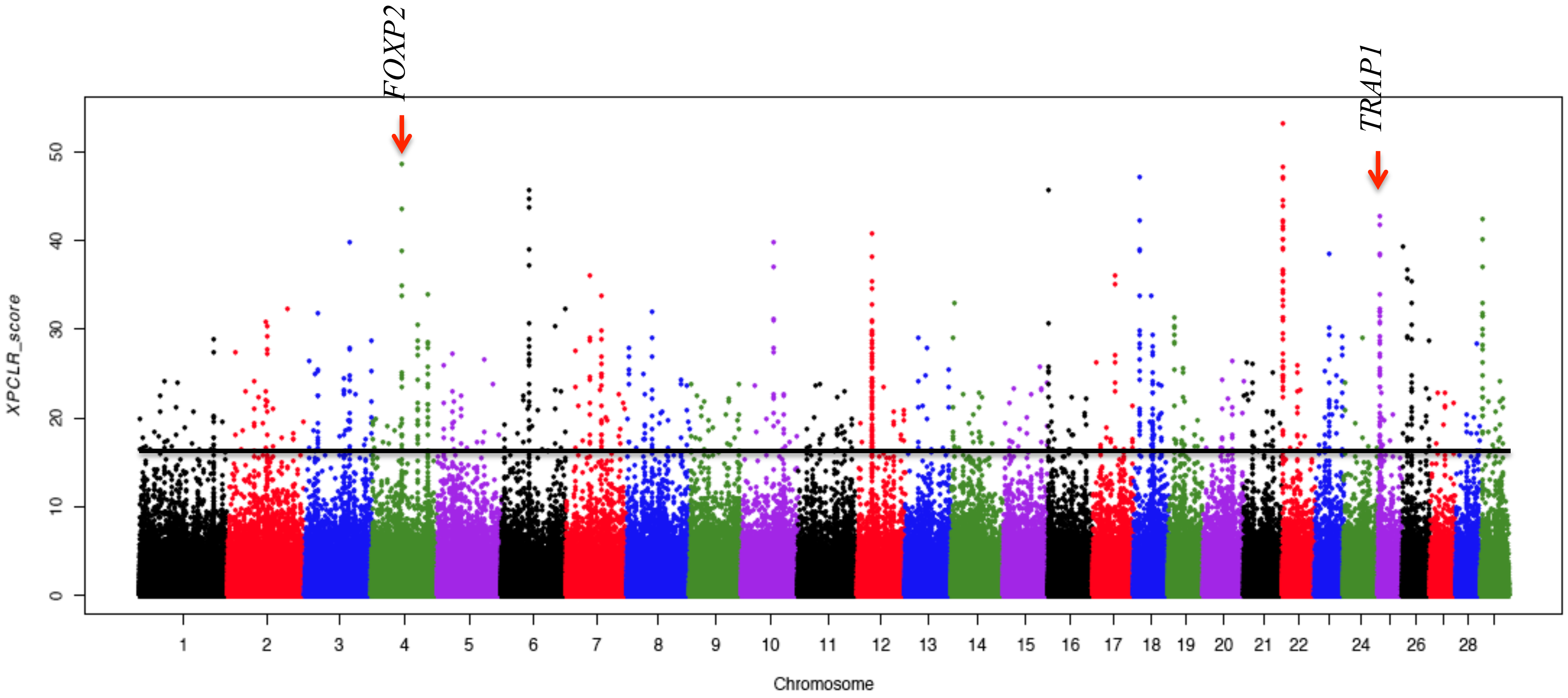

Figure S4: Plot of XP-CLR scores along autosomes in selective sweep analysis for the Northern goat population.

The horizontal line indicates a 0.1% autosomal-wide cut-off level. Red arrows and names indicate the two top candidate genes. The higher scores linked to the stronger signal on chromosome 22 were not associated to any annotated gene on the goat assembly (CHIR v1.0).
