# Supplementary material for: Characterizing neutral genomic diversity and selection signatures in indigenous populations of Moroccan goats (Capra hircus) using WGS data
Source: Front Genet. 2015 Apr 7;6:107. doi: 10.3389/fgene.2015.00107 (PMC4387958; doi:10.3389/fgene.2015.00107)
Supplement: Supplementary file 1 [file DataSheet1.ZIP › Supplemental Data/Table S1.docx]

**Table S1:** Characteristics of the 44 samples used for the analyses, their accession numbers in the Biosamples archive and the accession numbers of the sequencing data and aligned bam files in the ENA archive.

| Sample name | Biosample accession | ENA sequencing run | ENA aligned bam file | Estimated age (months) | Sex | Longitude (degrees) | Latitude (degrees) | Population |
| --- | --- | --- | --- | --- | --- | --- | --- | --- |
| MOCH-H19-1343 | SAMEA2012697 | ERR248929 | ERZ018783 | 12 | female | -11.05555 | +28.26907 | Draa |
| MOCH-K17-1351 | SAMEA2012705 | ERR315500 | ERZ018802 | 18 | male | -9.5984 | +29.02136 | Draa |
| MOCH-L17-1264 | SAMEA2012714 | ERR315508 | ERZ018745 | 36 | female | -9.25929 | +29.0379 | Draa |
| MOCH-L18-1280 | SAMEA2012707 | ERR234315 | ERZ018765 | 24 | male | -9.38272 | +28.50969 | Black |
| MOCH-N15-1209 | SAMEA2012756 | ERR234304 | ERZ018699 | 36 | female | -8.05893 | +30.26711 | Black |
| MOCH-N16-1228 | SAMEA2012757 | ERR234305 | ERZ018677 | 12 | female | -8.38965 | +29.60393 | Draa |
| MOCH-N16-1231 | SAMEA2012758 | ERR315503 | ERZ018727 | 36 | female | -8.08965 | +29.60393 | Draa |
| MOCH-N17-1237 | SAMEA2012759 | ERR315516 | ERZ018668 | 12 | male | -8.14666 | +29.2436 | Draa |
| MOCH-O14-1203 | SAMEA2012763 | ERR246143 | ERZ018741 | 36 | female | -7.52117 | +30.51695 | Black |
| MOCH-O16-1250 | SAMEA2012765 | ERR315510 | ERZ018743 | 36 | female | -7.56962 | +29.59379 | Black |
| MOCH-P14-1175 | SAMEA2012822 | ERR315498 | ERZ018763 | 18 | female | -7.08635 | +30.59224 | Draa |
| MOCH-P16-1251 | SAMEA2012823 | ERR246153 | ERZ018779 | 18 | female | -7.29038 | +29.51272 | Draa |
| MOCH-Q10-0090 | SAMEA2012826 | ERR229484 | ERZ018694 | >50 | female | -6.6516 | +32.7464 | Black |
| MOCH-Q11-0201 | SAMEA2012827 | ERR248933 | ERZ018806 | 24 | female | -6.5748 | +32.0533 | Black |
| MOCH-Q13-0153 | SAMEA2012829 | ERR229476 | ERZ018703 | . | male | -6.5553 | +31.0857 | Draa |
| MOCH-Q14-1167 | SAMEA2012830 | ERR315512 | ERZ018805 | >50 | female | -6.51941 | +30.52099 | Draa |
| MOCH-Q9-0208 | SAMEA2012834 | ERR248926 | ERZ018790 | 30 | female | -6.5208 | +33.2172 | Black |
| MOCH-R11-0005 | SAMEA2012835 | ERR229478 | ERZ018721 | . | female | -6.26669 | +32.22269 | Black |
| MOCH-R12-0195 | SAMEA2012836 | ERR229479 | ERZ018782 | 48 | female | -6.4226 | +31.649 | Black |
| MOCH-R13-1104 | SAMEA2012838 | ERR313264 | ERZ018770 | >50 | female | -6.02545 | +31.17796 | Draa |
| MOCH-R14-1105 | SAMEA2012839 | ERR248928 | ERZ018758 | 24 | female | -6.18389 | +30.56283 | Black |
| MOCH-R5-0037 | SAMEA2012891 | ERR340429 | ERZ018807 | . | female | -6.0898 | +35.201 | Northern |
| MOCH-R6-3007 | SAMEA2012892 | ERR315796 | ERZ018704 | 96 | female | -6.05 | +34.93 | Northern |
| MOCH-S12-1071 | SAMEA2012896 | ERR229471 | ERZ018693 | 12 | male | -5.5314 | +31.50069 | Black |
| MOCH-S13-1064 | SAMEA2012897 | ERR313261 | ERZ018675 | >50 | female | -5.52492 | +31.2501 | Black |
| MOCH-S15-1165 | SAMEA2012899 | ERR313254 | ERZ018757 | >50 | female | -5.55291 | +30.25376 | Draa |
| MOCH-S16-1135 | SAMEA2012900 | ERR234318 | ERZ018755 | 24 | female | -5.53758 | +29.59546 | Draa |
| MOCH-S4-0026 | SAMEA2012901 | ERR229473 | ERZ018710 | 60 | female | -5.87 | +35.73 | Northern |
| MOCH-S5-0045 | SAMEA2012902 | ERR219547 | ERZ018801 | >50 | female | -5.713 | +35.0314 | Northern |
| MOCH-T13-0128 | SAMEA2012898 | ERR340425 | ERZ018737 | >50 | female | -5.1657 | +31.1077 | Black |
| MOCH-T4-3026 | SAMEA2012911 | ERR345980 | ERZ018817 | 96 | female | -5.469 | +35.902 | Northern |
| MOCH-T5-0057 | SAMEA2012963 | ERR219544 | ERZ018689 | >50 | female | -5.2712 | +35.0991 | Northern |
| MOCH-T6-0074 | SAMEA2012964 | ERR219543 | ERZ018688 | 6 | male | -5.3537 | +34.9089 | Northern |
| MOCH-U11-1029 | SAMEA2012970 | ERR313258 | ERZ018774 | 12 | male | -4.59874 | +32.14459 | Black |
| MOCH-U13-1059 | SAMEA2012972 | ERR313268 | ERZ018723 | >50 | female | -4.52598 | +31.03844 | Draa |
| MOCH-U5-3014 | SAMEA2012974 | ERR332584 | ERZ018819 | 24 | female | -4.633 | +35.145 | Northern |
| MOCH-V8-2274 | SAMEA2013048 | ERR246139 | ERZ018730 | 96 | female | -4.45 | +33.8 | Black |
| MOCH-V9-1114 | SAMEA2013052 | ERR248923 | ERZ019216 | 24 | female | -4.10499 | +33.10143 | Black |
| MOCH-X10-2304 | SAMEA2013060 | ERR315795 | ERZ018822 | 4 | male | -3.416 | +32.9 | Black |
| MOCH-X6-2108 | SAMEA2013063 | ERR315782 | ERZ018754 | 72 | female | -3.16944 | +34.84444 | Black |
| MOCH-Z11-2197 | SAMEA2012106 | ERR332588 | ERZ018676 | 60 | female | -2.0833 | +32.4667 | Black |
| MOCH-Z6-2039 | SAMEA2012109 | ERR315513 | ERZ018669 | 48 | female | -2.1639 | +34.6431 | Black |
| MOCH-Z7-2010 | SAMEA2012110 | ERR248934 | ERZ018682 | 60 | female | -2.14917 | +34.02583 | Black |
| MOCH-Z9-2154 | SAMEA2037794 | ERR332576 | ERZ018687 | 12 | female | -2.0833 | +33.1333 | Black |
