# Supplementary material for: Characterizing neutral genomic diversity and selection signatures in indigenous populations of Moroccan goats (Capra hircus) using WGS data
Source: Front Genet. 2015 Apr 7;6:107. doi: 10.3389/fgene.2015.00107 (PMC4387958; doi:10.3389/fgene.2015.00107)
Supplement: Supplementary file 1 [file DataSheet1.ZIP › Supplemental Data/Table S2.docx]

**Table S2:** Summary of results from enrichment analysis for putative genes under selection in the Moroccan Black goat population.

| **GO term** | **Biological process** | **Number of genes associated** | **Number of candidate genes associated** | [**P-value**](http://cbl-gorilla.cs.technion.ac.il/GOrilla/z642mbzg/GOResultsPROCESS.html#p_value_info) | **Enrichment** |
| --- | --- | --- | --- | --- | --- |
| GO:0007070 | Negative regulation of transcription from RNA polymerase II promoter during mitosis | 2 | 2 | 8.42E-5 | 108.52 |
| GO:0007068 | Negative regulation of transcription during mitosis | 2 | 2 | 8.42E-5 | 108.52 |
| GO:0035295 | Tube development | 119 | 7 | 1.13E-4 | 6.38 |
| GO:0097091 | Synaptic vesicle clustering | 3 | 2 | 2.51E-4 | 72.35 |
| GO:0036444 | Calcium ion transmembrane import into mitochondrion | 3 | 2 | 2.51E-4 | 72.35 |
| GO:0097479 | Synaptic vesicle localization | 3 | 2 | 2.51E-4 | 72.35 |
| GO:0007389 | Pattern specification process | 231 | 9 | 2.81E-4 | 4.23 |
| GO:0042551 | Neuron maturation | 15 | 3 | 3.2E-4 | 21.70 |
| GO:0070542 | Response to fatty acid | 39 | 4 | 4.39E-4 | 11.13 |
| GO:0046021 | Regulation of transcription from RNA polymerase II promoter, mitotic | 4 | 2 | 4.99E-4 | 54.26 |
| GO:0048513 | Organ development | 829 | 18 | 5.58E-4 | 2.36 |
| GO:0009653 | Anatomical structure morphogenesis | 908 | 19 | 6.08E-4 | 2.27 |
| GO:0009887 | Organ morphogenesis | 263 | 9 | 7.21E-4 | 3.71 |
| GO:0045896 | Regulation of transcription during mitosis | 5 | 2 | 8.27E-4 | 43.41 |
| GO:0071398 | Cellular response to fatty acid | 21 | 3 | 8.99E-4 | 15.50 |
