# Supplementary material for: Characterizing neutral genomic diversity and selection signatures in indigenous populations of Moroccan goats (Capra hircus) using WGS data
Source: Front Genet. 2015 Apr 7;6:107. doi: 10.3389/fgene.2015.00107 (PMC4387958; doi:10.3389/fgene.2015.00107)
Supplement: Supplementary file 1 [file DataSheet1.ZIP › Supplemental Data/Table S3.docx]

**Table S3:** Summary of results from enrichment analysis for putative genes under selection in Draa goat population.

| **GO term** | **Biological process** | **Number of genes associated** | **Number of candidate genes associated** | [**P-value**](http://cbl-gorilla.cs.technion.ac.il/GOrilla/z642mbzg/GOResultsPROCESS.html#p_value_info) | **Enrichment** |
| --- | --- | --- | --- | --- | --- |
| GO:0001941 | Postsynaptic membrane organization | 5 | 3 | 1.26E-5 | 54.79 |
| GO:0071625 | Vocalization behaviour | 6 | 3 | 2.51E-5 | 45.66 |
| GO:0002087 | Regulation of respiratory gaseous exchange by neurological system process | 7 | 3 | 4.35E-5 | 39.14 |
| GO:0044065 | Regulation of respiratory system process | 8 | 3 | 6.91E-5 | 34.25 |
| GO:0060078 | Regulation of postsynaptic membrane potential | 41 | 5 | 8.00E-5 | 11.14 |
| GO:0060080 | Regulation of inhibitory postsynaptic membrane potential | 9 | 3 | 1.03E-4 | 30.44 |
| GO:0007158 | Neuron cell-cell adhesion | 10 | 3 | 1.46E-4 | 27.40 |
| GO:0030534 | Adult behavior | 108 | 7 | 1.8E-4 | 5.92 |
| GO:0044708 | Single-organism behavior | 279 | 11 | 2.46E-4 | 3.60 |
| GO:0043576 | Regulation of respiratory gaseous exchange | 12 | 3 | 2.63E-4 | 22.83 |
| GO:2000463 | Positive regulation of excitatory postsynaptic membrane potential | 12 | 3 | 2.63E-4 | 22.83 |
| GO:0035418 | Protein localization to synapse | 12 | 3 | 2.63E-4 | 22.83 |
| GO:0051899 | Membrane depolarization | 84 | 6 | 3.12E-4 | 6.52 |
| GO:0097113 | Alpha-amino-3-hydroxy-5-methyl-4-isoxazole propionate selective glutamate receptor clustering | 3 | 2 | 3.55E-4 | 60.88 |
| GO:0097104 | Postsynaptic membrane assembly | 3 | 2 | 3.55E-4 | 60.88 |
| GO:0072553 | Terminal button organization | 3 | 2 | 3.55E-4 | 60.88 |
| GO:0060079 | Regulation of excitatory postsynaptic membrane potential | 35 | 4 | 5.54E-4 | 10.44 |
| GO:0042391 | Regulation of membrane potential | 218 | 9 | 6.56E-4 | 3.77 |
| GO:0097119 | Postsynaptic density protein 95 clustering | 4 | 2 | 7.04E-4 | 45.66 |
| GO:0015727 | Lactate transport | 4 | 2 | 7.04E-4 | 45.66 |
| GO:0035873 | Lactate transmembrane transport | 4 | 2 | 7.04E-4 | 45.66 |
| GO:0051965 | Positive regulation of synapse assembly | 17 | 3 | 7.81E-4 | 16.12 |
| GO:0016337 | Single organismal cell-cell adhesion | 182 | 8 | 8.73E-4 | 4.01 |
| GO:0045838 | Positive regulation of membrane potential | 18 | 3 | 9.29E-4 | 15.22 |
| GO:0007610 | Behavior | 380 | 12 | 9.5E-4 | 2.88 |
