# Supplementary material for: Characterizing neutral genomic diversity and selection signatures in indigenous populations of Moroccan goats (Capra hircus) using WGS data
Source: Front Genet. 2015 Apr 7;6:107. doi: 10.3389/fgene.2015.00107 (PMC4387958; doi:10.3389/fgene.2015.00107)
Supplement: Supplementary file 1 [file DataSheet1.ZIP › Supplemental Data/Table S4.docx]

**Table S4:** Coat colors for the 14 Draa goats used in the analyses.

Colors were ordered according to their proportion in the individual coat.

| Sample name | Coat color |
| --- | --- |
| MOCH-U13-1059  MOCH-R13-1104  MOCH-S16-1135  MOCH-S15-1165  MOCH-Q14-1167  MOCH-P14-1175  MOCH-N16-1228  MOCH-N16-1231  MOCH-N17-1237  MOCH-P16-1251  MOCH-L17-1264  MOCH-H19-1343  MOCH-K17-1351  MOCH-Q13-0153 | Dark brown, Black, White  Light brown  Black, White, Light brown  White, Black, Dark brown  Dark brown, White, Black  Dark brown  White  White, Dark brown, Black  White, Light brown  Light brown  Light brown, White, Black  White, Light brown  Black  . |
