# Supplementary figures and images for: Characterizing neutral genomic diversity and selection signatures in indigenous populations of Moroccan goats (Capra hircus) using WGS data
Source: Front Genet. 2015 Apr 7;6:107. doi: 10.3389/fgene.2015.00107 (PMC4387958; doi:10.3389/fgene.2015.00107)

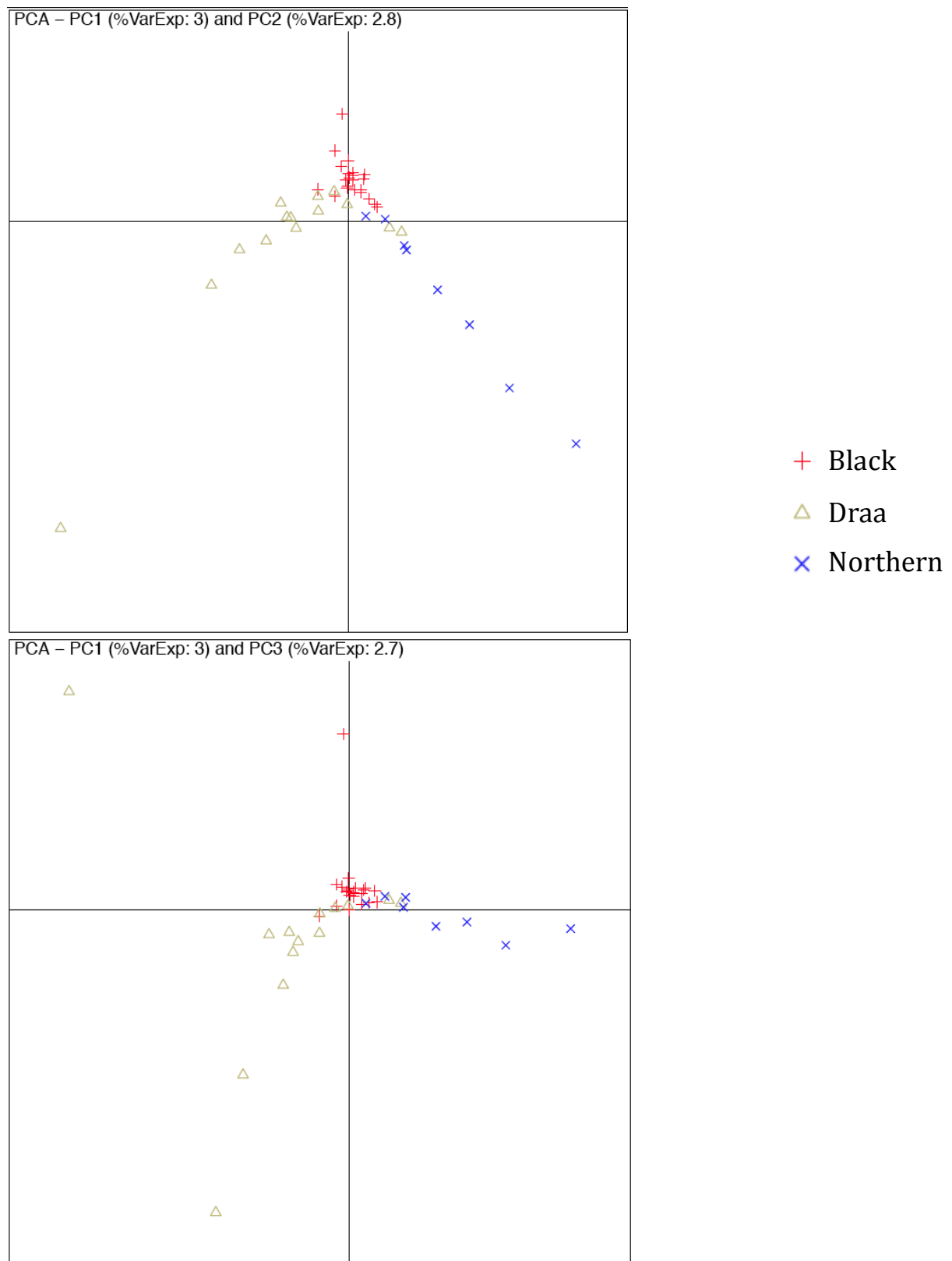

**Figure S1:** Principal Component Analysis based on the whole genome SNPs for the 44 Moroccan goats

Supplement: Supplementary file 1 [file DataSheet1.ZIP › Supplemental Data/Figure S1.pdf]
